# Using sounds for making decisions: greater tube-nosed bats prefer antagonistic calls over non-communicative sounds when feeding

**DOI:** 10.1242/bio.021865

**Published:** 2016-11-04

**Authors:** Tinglei Jiang, Zhenyu Long, Xin Ran, Xue Zhao, Fei Xu, Fuyuan Qiu, Jagmeet S. Kanwal, Jiang Feng

**Affiliations:** 1 Jilin Key Laboratory of Animal Resource Conservation and Utilization, Northeast Normal University, Jingyue St 2555, Changchun 130117, People's Republic of China; 2 Key Laboratory for Wetland Ecology and Vegetation Restoration of National Environmental Protection, Northeast Normal University, Jingyue St 2555, Changchun 130117, People's Republic of China; 3 Department of Neurology, Georgetown University, Washington, DC 20057, USA

**Keywords:** Acoustic communication, Social calls, Bats, Information, Decision making

## Abstract

Bats vocalize extensively within different social contexts. The type and extent of information conveyed via their vocalizations and their perceptual significance, however, remains controversial and difficult to assess. Greater tube-nosed bats, *Murina leucogaster*, emit calls consisting of long rectangular broadband noise burst (rBNBl) syllables during aggression between males. To experimentally test the behavioral impact of these sounds for feeding, we deployed an approach and place-preference paradigm. Two food trays were placed on opposite sides and within different acoustic microenvironments, created by sound playback, within a specially constructed tent. Specifically, we tested whether the presence of rBNBl sounds at a food source effectively deters the approach of male bats in comparison to echolocation sounds and white noise. In each case, contrary to our expectation, males preferred to feed at a location where rBNBl sounds were present. We propose that the species-specific rBNBl provides contextual information, not present within non-communicative sounds, to facilitate approach towards a food source.

## Abstract (precis)

**Summary:** In this study we used sound playback to test the importance of communication sounds of bats as contextual information for decision making for place preference during feeding.

## INTRODUCTION

Communication signals play a key role in social interactions and are especially important when senders and receivers have a conflict of interest, such as competing for mate, food and space (Irschick et al., 2014). In bats, which are highly vocal, communication sounds are the primary mediators of social interactions. They typically live and interact socially with conspecifics either under low-light or in complete darkness. They use echolocation signals to locate, navigate and forage (Kunz and Fenton, 2003). For social interactions with conspecifics, many species of bats emit complex sounds, which are hierarchically organized as ‘syllables’, ‘composites’ and ‘trains’ within or as calls (Kanwal et al., 1994) that serve a communicative function.

*M. leucogaster* use a wide variety of communication sounds within different social contexts, including 12 simple syllables and five composites (Lin et al., 2015). Males of *M. leucogaster* engage in agonistic interactions and emit harsh and relatively low-frequency calls when feeding freely in a captive environment, presumably to deter the threat posed by intruders, and also within other contexts, e.g. to ward off male intruders from approaching and entering a colony (Lin et al., 2015). Previous studies in this and other species have focused on defining the behavioral significance and acoustic characteristics of calls in bats (Behr and von Helversen, 2004; Bohn et al., 2008; Clement and Kanwal, 2012; Gadziola et al., 2012; Kanwal et al., 1994; Leippert, 1994; Lin et al., 2015; Luo et al., 2006; Ma et al., 2006; Wright et al., 2013). Playback experiments, however, can provide deeper insights into the adaptive and communicative role of social calls, but have been rarely conducted to test call perception in bats (Barlow and Jones, 1997; Corcoran and Conner, 2014; Eckenweber and Knörnschild, 2016; Fernandez et al., 2014).

Agonistic calls emitted by *M. leucogaster* include the noise burst (NB) element, and spectrographically match the previously classified long, rectangular broadband noise burst (rBNBl) call type (Lin et al., 2015). In this study, we used a two-choice, place-preference experimental design to assess the function of rBNBl in greater tube-nosed bats. We simultaneously played back either rBNBl versus echolocation sounds, or rBNBl versus white noise. A hypothesis of no differences would suggest that bats do not exhibit any preference. Furthermore, we hypothesized that being an aggressive call type, as shown in this (Lin et al., 2015) and other bat species (Clement et al., 2006), rBNBl would act as a deterrent, driving the preference of the males towards the alternate food source. Our results, however, indicated otherwise, suggesting alternate motivational and/or context-sensitive mechanisms at play that may underlie alternate perception/s of the same communication sound.

## RESULTS AND DISCUSSION

**Fig. 1.**
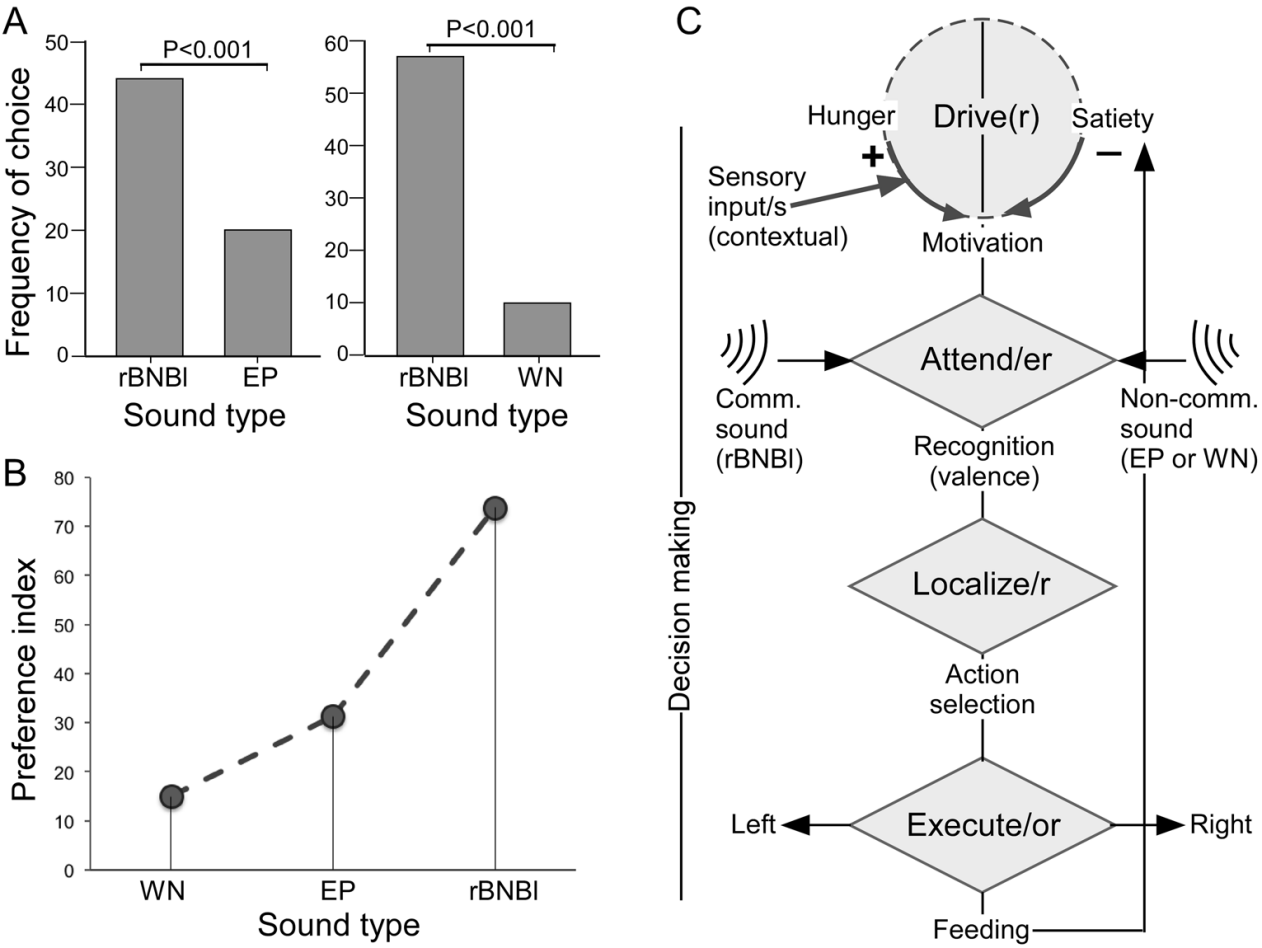
**Preference for different types of sound stimuli and decision making process.** (A) Bar graphs showing frequency of choices as demonstrated by *Murina leucogaster* between long broadband noise bursts and echolocation sounds, and between long broadband noise bursts and white noise. (B) Plot of relative values of the preference index for different types of sound stimuli as attractors/distracters influencing place of feeding. (C) Flow chart showed sequence of physiological states and mechanisms influencing decision making for two-choice place preference in response to playback of sounds during presentation of food. The observed behavior is considered to consist of the ‘Drive-Attend-Localize-Execute’ sequence with the involvement of the appropriate neural systems. Shaded circles and diamonds represent generalized brain mechanisms/circuits that participate in the decision process within a specific context and emotive state. Motivational drivers consist of the hunger and satiety centers within the hypothalamus (Morgane, 1961). ‘Hunger neurons’ suppress activity in the satiety center in the hypothalamus, a reciprocal interaction between hunger and satiety centers in the mammalian brain (hypothalamus) is well established (Morgane, 1961). After recognizing the sound and its meaning, neural computations are needed to localize the source of sounds of interest (Fuzessery and Pollak, 1984). The valence of stimuli is evaluated and provided by structures such as the amygdala and nucleus accumbens within the limbic forebrain (Namburi et al., 2016), which receive information processed by the auditory system and bias the motor circuits (within cortex, basal ganglia and/or brainstem) deciding on the place to feed. EP, echolocation pulses; rBNBl, long rectangular noise burst syllable; WN, white noise.

In the first two-choice experiment, 64 of the 71 trials were successful. Of these, in 44 trials (nearly 70%), bats selected the rBNBl side for feeding. In the remaining 20 trials (∼30%), animals preferred to approach and feed on the echolocation playback side. Preference for the rBNBl playback side was highly significant (*P*=0.00369) (Fig. 1A).

At the individual level, each bat was tested three times, and 17 bats successfully completed all three trials. About a third (*n*=5) of the total number of individuals selected the food dish on the rBNBl side for each of the three trials. Nine of them selected the rBNBl side twice as their preferred feeding site. These results indicate that 14 individuals (∼82% of total individuals) displayed a high probability for exposure to rBNBl while feeding (*P*=0.013). The other three individuals (about 18% of total individuals) chose to completely avoid approaching/feeding on the rBNBl side.

In the second two-choice experiment, 67 of the 72 trials were successful; the remaining were scored as mistrials. Our results show that *M. leucogaster* approached and fed near rBNBl in 57 trials (∼85%). In another 10 trials (∼15%), individuals preferred the acoustic environment of white noise when feeding. The preference for rBNBl over white noise was significant (*P*=4.04E-9) (Fig. 1A). At the individual level, each bat was tested three times and 21 bats successfully chose to feed all three times; of these, 13 individuals preferred the place of rBNBl playback all of the three times. Seven individuals twice selected the rBNBl side to feed. These results show that, except for one individual, all others (∼95% of total individuals) displayed a ≥67% probability to select the food dish at the source of rBNBl sounds (*P*=0.000021). Overall, males showed the highest preference for rBNBl and were clearly not deterred by this antagonistic sound (Fig. 1B). Echolocation sounds were preferred at a rate (preference index=% of animals choosing a particular sound) that was more than twice as high as that for white noise.

In bats, as in many other species, antagonistic calls are emitted when males fight with each other either to gain/maintain territory (roosting location) or when competing for females within the bat colony. Therefore, the expectation was that the bats would avoid feeding in the presence of rBNBl. Our results showed otherwise. Male bats, in fact, approached the food trays where the ambient acoustic environment consisted of rBNBl, an antagonistic call. Below we present a common mechanistic framework (Fig. 1C) within which to consider and interpret our results.

Given what we already know about the brain and behavior in mammals, the decision to choose where to feed is the outcome of a series of existing conditions and processes. First, the animal has to be in the physiological/mental state to drive the feeding behavior. Since bats were food deprived, they were hungry and motivated to feed; and therefore, hunger primes attention (LaBar et al., 2001; Mohanty et al., 2008). Although not explicitly tested here, we presume that release of the observed feeding behavior, triggered by the presentation of auditory cues, was mediated by attentional mechanisms. These mechanisms constitute the first two steps that ‘drive’ the behavior and facilitate animals to ‘attend’ to the task, respectively, in the flowchart depicted in Fig. 1C.

Similar to grasping real world objects using eye fixations in humans (Belardinelli et al., 2015), we presume that perception of the antagonistic rBNBl call facilitated fixation of their attention on the appropriate side. This helped bats to obtain additional contextual information gleaned from recognizing and evaluating the two types of playback sounds to determine the appropriate course of action. It is possible that in the absence of meaningful echolocation vocalizations, bats perceived agonistic interactions as evidence for the presence of high-quality food and preferred to fight it out for food rather than stay hungry. According to studies in birds and mammals, including nonhuman primates, food-associated calls may communicate the caller's level of excitement or arousal in response to the feeding event (Clay et al., 2012; Owren and Rendall, 2001). Echolocation vocalizations are designed primarily to facilitate foraging and for locating objects in dark environment (Melcón and Moss, 2014).

Sound localization circuits are well-studied in the midbrain of bats and other species (e.g. Fuzessery and Pollak, 1984). In our experiment, localizing the direction from which the preferred sound is emanating is a key step in the decision-making process (Fig. 1C). We label this as the ‘localize’ step in our flowchart. A decision is not of much use if it is not executed appropriately, and execution involves integration of all of the above information followed by action sequence. We therefore label ‘execute’ as the final step in the decision-making process shown in our flowchart, which leads to the feeding behavior observed and tested in this study.

We note that echolocation sounds can also act as sources of information regarding the location and possibly the quality of food (Jones and Siemers, 2011). Since echolocation pulses emitted by conspecifics are familiar, they may even have a calming effect on conspecifics (Li et al., 2014; Schuchmann and Siemers, 2010). For these reasons as well, we assumed that bats would feed on the side where echolocation sounds were being played back. However, in only 20 trials, *M. leucogaster* selected the food dish on the side with continuous playback of echolocation sounds. We presume the randomly selected echolocation sounds neither motivated nor conveyed any relevant information about the food.

White noise playback showed the least preference, likely because broadband noise can potentially interfere with the bats' own echolocation signals for ‘visualizing’ the food. In fact, ambient noise generated at loud compressor sites is known to suppress activity levels for *Tadarida brasiliensis* (Bunkley et al., 2015). *Myotis myotis* also avoids foraging in areas with particularly loud background noise (Schaub et al., 2008). Therefore, it is conceivable that white noise interfered with or jammed echolocation signals, making it difficult for *M. leucogaster* to choose and/or track their prey. Our experimental design has direct implications for foraging decisions made by bats in the field and the potential for environmental noise pollution to degrade or delay important communication or sound-triggered decision making in the wild.

In this study, relative to echolocation sounds and white noise, male bats preferred to feed in the vicinity of rBNBl playback, which implies that rBNBl may contain some information that interested the bats. The rBNBl sound is produced in a situation when two individuals of *M. leucogaster* contest with or defend their food resource (Lin et al., 2015). This most likely corresponds to the signaler's motivational response (e.g. excitement or arousal) to a mealworm in the food dish. However, antagonist interactions may also occur when individuals fight for the same high-quality food. Such interactions have been observed at mealworm food trays provided to a colony of captive bats (J. S. K., unpublished data). To further test the behavioral model, rBNBl sounds could be switched either to white noise or to echolocation sounds once the animal initiates approach. This will test the extent to which the initial localization and approach decisions postulated in our model are reversible.

In conclusion, we provide evidence in *M. leucogaster* for their ability to extract significant contextual information from complex communication sounds. Our literature review and observations suggested that the rBNBl side was preferred for feeding because it attracted attention, being associated with antagonistic interactions during feeding. These findings open the door for conducting additional playback experiments as a method to better understand the role that communication sounds play in the social life of bats. More specifically, the fact that the majority of males preferred to feed in the vicinity of sounds associated with agonistic interactions, suggests that rBNBl sounds can play an indirect role as a source of contextual information to attract conspeciﬁcs in addition to serving a more direct function as a deterrent during antagonistic interactions. Clearly, more experiments that include manipulation of hormonal levels in the tested individuals are needed to further validate our model and fully understand the functional complexity of communication sounds and the varied roles they play in the social life of bats.

## MATERIALS AND METHODS

### Acquisition and maintenance of animals

Twenty-five adult male greater tube-nosed bats, *M. leucogaster* were captured in April 2015 from Dalazi cave (125°50′9.9″ E, 41°3′55.8″ N) in Ji'an, Jilin province, China. Bats were marked and housed in a free-flight husbandry room (8 m×5 m×3 m) maintained at 22°C, 60% humidity and 12 h light:12 h dark cycle. Food and water dishes were set up on a tray mounted on the wall of the room. The bats were fed on a diet of mealworms *ad libitum*, enriched with vitamin and mineral supplements. We cleaned the ﬂoor, walls, food and water dishes daily to ensure the health of the animals. All procedures were in accordance with National Natural Science Foundation of China for experiments involving vertebrate animals and were approved by Northeast Normal University Animal Research Committee.

### Sound selection, synthesis and conditioning

**Fig. 2.**
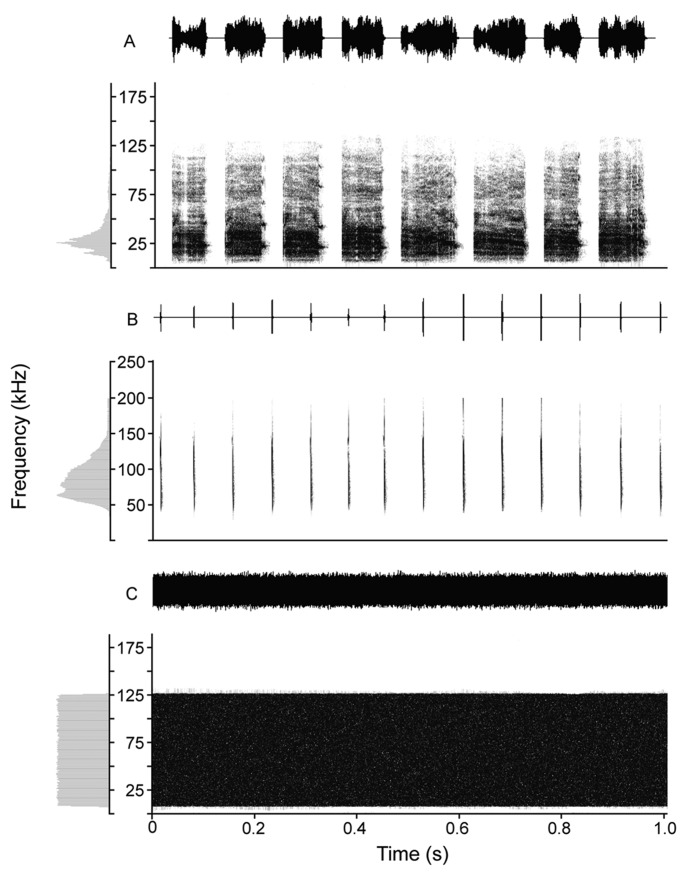
**The playback stimuli.** The energy spectrum (left), oscillogram (top) and spectrogram (bottom) of long broadband noise bursts (A), echolocation sounds (B) emitted by *Murina leucogaster*, respectively, and digitally generated white noise (C).

We randomly selected the highest quality rBNBl syllables from the digitized library of calls from which we synthesized a sequence of syllables that were all together one minute in duration (Fig. 2A). Individual rBNBl syllables present in calls emitted by greater tube-nosed bats during agonistic interactions have an average duration of 100.43±25.16 ms (*n*=13). A noise-filled rectangular shape characterized the spectrogram of each syllable. The mean predominant frequency of rBNBl syllables was 27.25±2.51 kHz (*n*=13). Similarly, we selected echolocation pulses or sounds, emitted during the search phase, to synthesize a one-minute sound file of *M. leucogaster* (Fig. 2B). In addition, a one-minute sound file of white noise with the same bandwidth to rBNBl (5–125 kHz) was synthesized using Avisoft–SASLab Pro (Avisoft Bioacoustics, Berlin, Germany) (Fig. 2C). All sound samples were normalized to 75% (RMS value) of the maximum recorded amplitude using Avisoft –SASLab Pro.

### Experimental setup and design

**Fig. 3.**
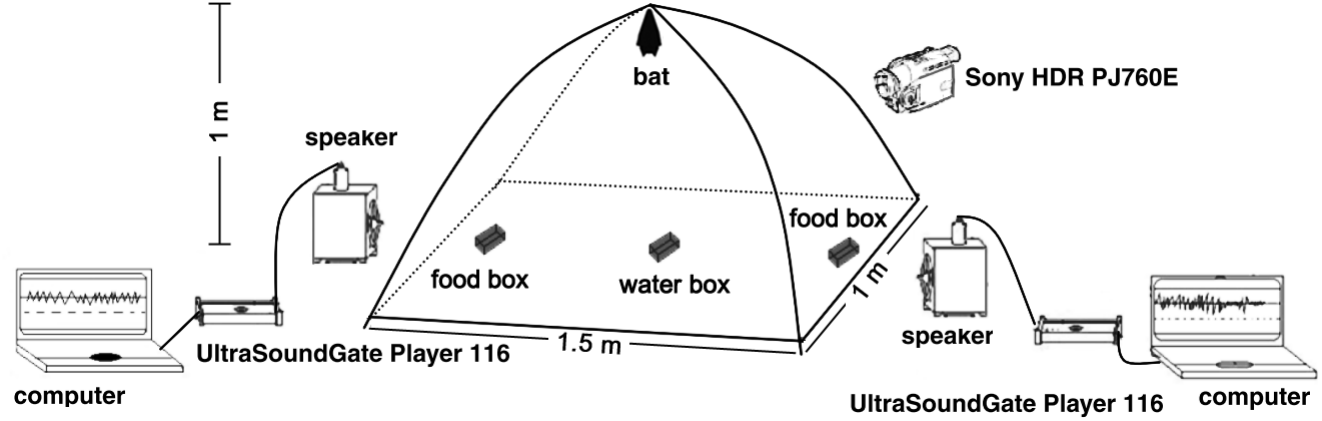
Illustration of experiment set up for the two-choice experiments in *Murina leucogaster*. See the details in Materials and methods section in main text.

A tent (1.5 m×1 m×1 m, Fig. 3) built from mosquito netting and placed in a large, echo-attenuated (by up to 50 dB) recording room (5 m×2 m×3 m) in a relatively quiet location in the basement of the building was used to perform the two-choice experiments. We placed a food dish on each side at the bottom and a water dish on the floor in the middle of the tent. An UltraSound Gate Player 116 with a speaker on each side was placed outside of tent, 20 cm away from the food dish. An infrared digital video camera (Sony Handycam HDR-PJ760E, Japan) was used to monitor the bat's response.

Bats were divided into two groups (12 and 13 individuals, respectively). A bat from each group was tested on alternate days. This cycle was repeated until all bats had been tested. Bats were allowed to hang at the center of the tent (their preferred resting location being the highest location inside the tent) for an hour prior to initiating the trial to allow them to acclimate. In each playback trial, each bat was typically tested three times. To increase their motivation to feed, bats were food deprived for 24 h prior to initiating the two-choice experiment. The same number of mealworms was placed in each of the two food dishes before initiating playback. The room was kept in complete darkness and experimenters avoided making any noise to minimize disturbance that could bias the data. All of the two-choice, place-preference tests were performed during the bats' daily peak activity period (14:00-17:00) in their captive environment.

In the first two-choice trial, rBNBl and echolocation sounds were played back simultaneously to the bat. Since each bat was tested three times on different days, we alternated the playback of the two sounds between the two sides to exclude learned auditory bias between trials. A trial was considered successful if a bat approached a food dish and commenced eating mealworms within 5 min. The trial was scored a mistrial if a bat did not approach and feed from either of the two trays within 5 min. In the second two-choice experiment, playback of echolocation sounds was replaced with white noise; other experimental procedures were kept the same as the first experiment.

### Statistical analysis

We used the exact binomial test of goodness-of-fit to analyze significant differences in frequency of choice for both sides for all experimental trials (*P*<0.05). In addition, we also assessed the differences in frequency of choice for each side at the individual level, using the same test.
